# CD8^+^ Tumour-Infiltrating Lymphocytes and Tumour Microenvironment Immune Types as Biomarkers for Immunotherapy in Sinonasal Intestinal-Type Adenocarcinoma

**DOI:** 10.3390/vaccines8020202

**Published:** 2020-04-28

**Authors:** Rocío García-Marín, Sara Reda, Cristina Riobello, Virginia N. Cabal, Laura Suárez-Fernández, Blanca Vivanco, Fernando López, José L. Llorente, Mario A. Hermsen

**Affiliations:** 1 Department Head and Neck Oncology, Instituto de Investigación Sanitaria del Principado de Asturias, 33011 Oviedo, Spain; rociogm220879@hotmail.com (R.G.-M.); cristinarisu15@gmail.com (C.R.); vircabal@hotmail.com (V.N.C.); laura_quillo@hotmail.com (L.S.-F.); 2 Department Otolaryngology, Hospital Universitario Central de Asturias, 33011 Oviedo, Spain; sara_reda_91@hotmail.com (S.R.); flopez_1981@yahoo.es (F.L.); llorentependas@telefonica.net (J.L.L.); 3 Department Pathology, Hospital Universitario Central de Asturias, 33011 Oviedo, Spain; vivancoblanca@gmail.com

**Keywords:** sinonasal cancer, intestinal-type adenocarcinoma, CD8^+^ TILs, tumour microenvironment immune type, immunotherapy

## Abstract

Background. Intestinal-type adenocarcinoma (ITAC) is a rare tumour occurring in the ethmoid sinus. Recent years have brought advances in endoscopic surgery and precision radiotherapy; however, five-year overall survival has not improved and remains at 35–80%, depending on tumour stage and histology. Therefore, there is a need for new therapeutic options. Methods. We evaluated CD8^+^ tumour-infiltrating lymphocytes (TILs) and tumour microenvironment immune type (TMIT, combining CD8^+^ TILs and PD-L1) as predictive biomarkers for immunotherapy in a series of 133 ITAC. All results were correlated to clinical and follow-up data. Results. The presence of intratumoural CD8^+^ TILs was low in 57% of cases and high in 8% of cases. Tumoural PD-L1 positivity was observed in 26% of cases. CD8^+^ TILs and TMIT correlated with the histological subtype of ITAC and with better overall survival. The presence of stromal PD-L1-positive macrophages was related to intratumoural CD8^+^ TILs. PD-L1 expression on tumour cells or macrophages did not show prognostic value. Conclusions. TMIT classification did not have additional prognostic value over CD8^+^ TILs alone. The modest percentage of CD8^high^/PD-L1^pos^ cases indicates that ITAC is a lowly immunogenic tumour type. Nevertheless, a proportion of ITAC, especially the papillary and colonic subtypes, could benefit from therapy with immune checkpoint inhibitors.

## 1. Introduction

Intestinal-type adenocarcinoma (ITAC) is a rare tumour, with an approximate incidence of less than one case per 100,000 inhabitants annually, accounting for 10–20% of all tumours in the sinonasal area, although these proportions vary geographically [1,2,3]. ITAC is etiologically related to professional exposure to organic dust, mainly wood or leather [4,5,6]. Due to the lack of specific symptoms at early stages, patients are often diagnosed at advanced stages. ITAC can be classified into four subtypes, i.e., papillary, colonic, solid and mucinous, the latter two having a worse clinical behavior [7]. 

The main treatment modality is surgery with postoperative radiotherapy, and in some cases combined modalities including chemotherapy may be used. Surgical treatment has advanced due to the progressive application of transnasal endoscopic techniques for naso-ethmoidal malignancies [4,8,9,10,11]. However, patients with ITAC still face an unfavourable prognosis, with a five-year survival rate ranging from 35% for stage IV to 80% for stage I tumours [2,4,8,9,10,11,12]. Local recurrence often occurs within two years of follow-up and is the main contributor to sinonasal cancer mortality. Therefore, new treatment options for neoadjuvant, concomitant o adjuvant therapy are needed. Immunotherapy, especially immune checkpoint inhibitors, may be such an option.

Several monoclonal antibodies have been developed that interfere with signalling pathways used by tumours to evade the immune system. Programmed death-ligand 1 (PD-L1) is expressed on the cell surface of tumour cells, as well as B and T lymphocytes, macrophages, and dendritic cells. The interaction between PD-L1 on tumour cells and the programmed cell death protein 1 (PD-1) receptor on activated cytotoxic T cells inhibits the immune response mediated by interferon (IFN), reducing the cytotoxic activity of T cells, and protects from apoptosis [13]. The results of several clinical trials have led to the approval of anti-PD-1 agents, such as Pembrolizumab and Nivolumab, for a number of tumour types including head and neck squamous cell carcinoma (HNSCC) [14,15,16]. However, only a proportion of patients respond to the treatment, so there is a need to identify predictive biomarkers. 

Most studies have concentrated on tumour PD-L1 expression and the presence of various tumour-infiltrating lymphocytes (TILs). In a previous study, our group reported 17% of tumours with PD-L1 expression in a series of 126 ITAC [17], indicating a subgroup of patients that may benefit from anti-PD-1 therapy. Although possibly a predictive marker for immunotherapy, PD-L1 positivity did not have prognostic value. We are not aware of published studies on ITAC similar to ours; however, there are reports on PD-L1- and CD8-positive TILs (CD8^+^ TILs) in other sinonasal tumour types. Approximately 30–35% of sinonasal squamous cell carcinomas (SNSCC) expressed PD-L1 [17,18], while CD8^+^ and CD4-FOXP3 TILs were detected in 50% of cases and correlated with better survival [18]. CD8^+^ TILs were also related to better survival in olfactory neuroblastoma [19]. A classification based on both TILs and tumour PD-L1 expression, named tumour microenvironment immune type (TMIT), recognizes four immunotypes: type I (TIL^high^/PD-L1^pos^), type II (TIL^low^/PD-L1^neg^), type III (TIL^low^/ PD-L1^pos^) and type IV (TIL^high^/ PD-L1^neg^) [20]. Later, TILs evaluated in TMIT became specified as CD8^+^ TILs; their correlation to features like high mutational burden and oncogenic viral infection supported the clinical relevance of this classification [21]. TMIT does not reflect the tumour microenvironment which includes many types of TILs, however, it may predict the response to immunotherapy on an individual basis, which is reported to be an important marker when selecting combined treatments for cancer [22,23,24].

The aim of this study was to evaluate the prevalence of CD8^+^ TILs and TMIT in a large series of ITAC as possible predictors of response to immunotherapy. In addition, all results were correlated with clinico-pathological and follow-up data. 

## 2. Materials and Methods

### 2.1. Patients and Samples

Samples from 133 previously untreated ITAC patients treated between 1978 and 2014 were collected from the biobank archives of our hospital. All patients had signed an informed consent for the collection, analysis and storage of their biological material, and the study was approved by the Institutional Ethics Committee of the Hospital Universitario Central de Asturias and by the Regional CEIC from Principado de Asturias (approval number 07/16 for project CICPF16008HERM). The mean patient age was 66 years, and 98% (131/133) of patients were males. The distribution of disease stage according to the TNM system for tumour classification [25] was: 30 tumours in stage I, 17 in stage II, 45 in stage III, 16 in stage IVa and 25 in stage IVb. Thirteen cases were papillary, 80 were colonic, 10 were solid, and 30 were mucinous histological subtype [7]. A history of professional exposure to wood dust was recorded for 118 of 133 (89%) patients. Seventy-six (57%) patients received radiotherapy after radical surgery. The median follow-up was 56 months (range 1–460). Details on the clinical features are presented in Table 1.

### 2.2. Immunohistochemistry

Tissue microarray (TMA) blocks were prepared from formalin-fixed, paraffin-embedded tumour tissues using the Beecher Tissue Microarrayer (Beecher Instruments, Silver Spring, MD, USA). In total, 5 TMA blocks were constructed, containing three 1 mm cores from different areas of 133 ITAC tumours. Immunohistochemistry (IHQ) was performed on an automatic staining workstation (Dako Autostainer Plus; DakoCytomation, Glostrup, Denmark). Antibodies anti-PD-L1 clone E1L3N (1/100 monoclonal rabbit, Cell Signalling Technology, Cambridge UK), anti-CD8 clone C8/144B, IR623 (Prediluted monoclonal mouse, DAKO, Glostrup, Denmark), were applied using high-pH antigen retrieval for 20 min. The slides were evaluated in a double-blind manner by three observers. The staining was visualized by light microscopy.

CD8^+^ TILs were scored as 0%, 1–10% and >10% of the cells present in the stromal or in the intratumoural compartment. Staining intensity was similar in all tumours. Staining for PD-L1 was considered positive when >5% of the tumour cells showed membranous and/or cytoplasmic staining. TMIT subtypes were defined by the combination of intratumoural CD8^+^ TIL presence (CD8^low^, either 0% or 1–10%, and CD8^high^, >10%) and PD-L1-stained tumour cells (negative, <5% and positive, >5%). TMIT type I was defined as CD8^high^/PD-L1^pos^, type II as CD8^low^/PD-L1^neg^, type III as CD8^low^/PD-L1^pos^ and type IV as CD8^high^/PD-L1^neg^. In the stromal compartment, we observed PD-L1-stained macrophages, which were scored as either absent or present.

### 2.3. Statistical Analysis

The Chi^2^ test was used to test possible associations between CD8^+^ TILs, PD-L1 expression, TMIT types and various clinicopathological factors. Kaplan–Meier curves were plotted to assess the relations of CD8^+^ TILs and TMITs to overall survival, using the log-rank-test; *p*-values < 0.05 were considered to indicate statistical significance. Multivariate Cox regression analysis was performed for factors possibly related to survival. Statistical analysis was carried out with the use of SPSS Base, version 15.0 and SPSS Advanced models, version 15.0 (SPSS Inc., Chicago, IL, USA) software.

## 3. Results

### 3.1. Tumour Stage and Histological Subtype Are Related to Longer Survival

**Figure 1:**
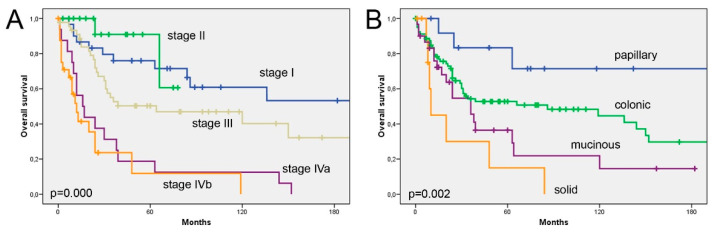
Overall survival according to tumour stage (**A**) and histological subtype (**B**).

During the period of follow-up, 63 of 133 patients (48%) developed local recurrence, and 13 (10%) distant metastasis. Sixty of 133 patients (45%) remained alive, and the median disease-free time was 12 months (range 1–96 months). (Table 1). The overall five-year survival was 50%, the main causes of death being local recurrence and intracranial invasion; however, 20 patients died during the postoperative period or due to intercurrent causes. Overall survival (Figure 1) was significantly related to disease stage (log-rank 50.498; *p* = 0.000) and histological subtype (log-rank 15.085; *p* = 0.002).

### 3.2. The Prognostic Value of CD8^+^ TILs Is Independent of Tumour Stage and Histological Subtype

**Figure 2:**
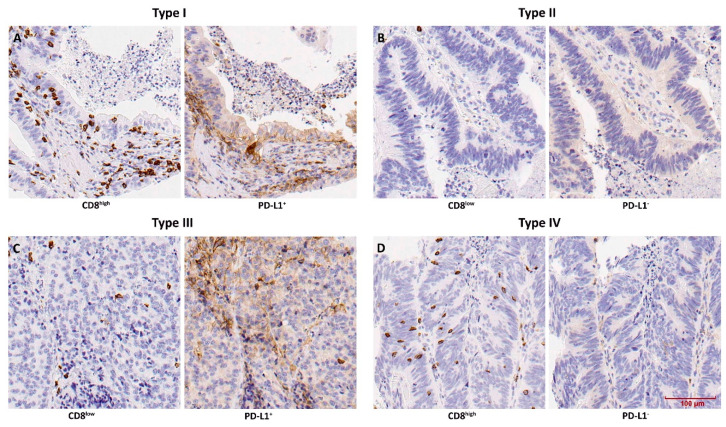
Immunohistochemical staining of CD8^+^ TILs and PD-L1-expressing tumour cells, in the four TMIT types: (**A**) type I, (**B**) typeII, (**C**) type III and (**D**) type IV.

**Figure 3:**
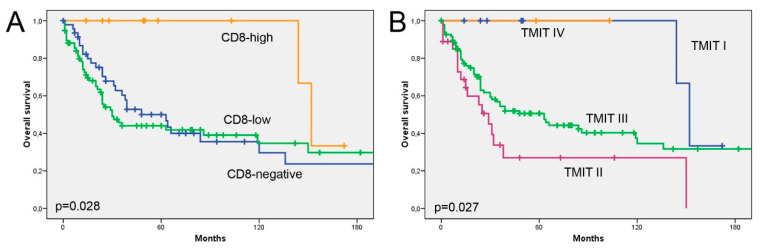
Overall survival according to CD8^+^ TILs (**A**) and TMIT (**B**).

Stromal CD8^+^ TILs were detected in the majority (126/133, 95%), and intratumoural CD8^+^ TILs in 65% (86/133) of cases. All samples with intratumoural CD8^+^ TILs also showed stromal CD8^+^ TILs, reason why we focused our analyses on intratumoural CD8^+^ TILs only. Complete absence of CD8^+^ TILs was seen in 35% (47/133) of samples. The presence of intratumoural CD8^+^ TILs was low in 57% of cases and high in 8% of cases (Figure 2). All (10/10) CD8^high^ cases were histologically papillary and colonic, and also among the CD8^low^ cases these ITAC subtypes were dominant (75%, 57/76). CD8^high^ cases also appeared to be at a lower tumour stage and developed less recurrence or metastasis compared to CD8^low^ and CD8^neg^ tumours (Table 1). Kaplan–Meier analysis showed that CD8^high^ cases had significantly better overall survival than CD8^low^/CD8^neg^ cases (*p* = 0.028) (Figure 3). Using multivariate Cox regression, we found that presence of CD8^+^ TILs >10% was a positive prognostic factor independent of tumour stage, while histological subtype lost its prognostic value due to its interrelation with CD8^+^ TILs (Table 2). 

### 3.3. TMIT Types Display Different Clinical Outcomes

The most frequent TMIT was type II, observed in 72% (96/133) of cases, followed by type III in 20% (27/133), type I in 6% (8/133) and type IV in 1% (2/133) of cases. No significant correlation was found between TMIT and any of the clinicopathological characteristics (Table 1). Kaplan–Meier analysis showed that type I and IV cases (CD8^high^ and either PD-L1^pos^ or PD-L1^neg^) had much better overall survival (LR 9.186, *p* = 0.027). Analysis of only TMIT II and III cases (CD8^low^ and either PD-L1^pos^ or PD-L1^neg^) revealed a worse overall survival for those tumours expressing PD-L1, although not statistically significant (LR 3.593, *p* = 0.058) (Figure 3).

### 3.4. PD-L1-Expressing Tumour Cells and Macrophages Co-Occur with CD8^+^ TILs

**Figure 4:**
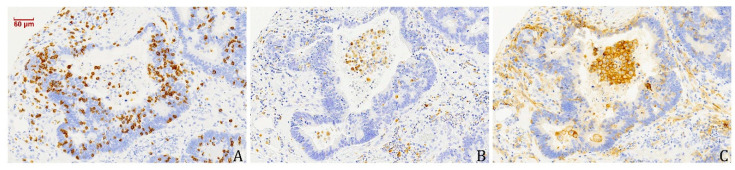
Immunohistochemical staining of CD8^+^ TILs (**A**) and CD68- (**B**) and PD-L1- (**C**) expressing macrophages.

By analysing PD-L1 staining for the evaluation of TMIT, we found 26% (35/133) of cases with positivity in patches of tumour cells, and this was positively correlated (*p* = 0.000) with intratumoural presence of CD8^+^ TILs (Table 3). We also observed a notable PD-L1 positivity on macrophages, particularly in the stromal compartment (Figure 4), again correlated (*p* = 0.001) with intratumoural presence of CD8^+^ TILs (Table 3). Mucinous ITAC displayed a notable lower presence of PD-L1-positive macrophages compared to papillary, colonic and solid ITAC, corresponding, respectively, to 3% and 18–30%. We found no other correlations between the presence of PD-L1-positive macrophages and clinical and follow-up data (Table S1). 

## 4. Discussion

Patients with ITAC in advanced stages generally have a poor prognosis, with limited therapeutic options. In this study, we analysed 133 ITAC for the presence of CD8^+^ TILs and TMIT, combining the presence of CD8^+^ TILs and the expression of PD-L1 on tumour cells as a possible predictive marker for treatment with PD-1/PD-L1 immune checkpoint inhibitors. In addition, we investigated their possible prognostic value. To the best of our knowledge, this is the first study analysing CD8^+^ TILs and TMIT as indicators for immunotherapy in sinonasal ITAC.

We found 65% (86/133) of cases with intratumoural CD8^+^ TILs, all of which also showing these lymphocytes in the stromal compartment. Kaplan–Meier survival analysis revealed no difference between cases with absence and low presence of CD8^+^ TILs; however, all cases with a high presence of CD8^+^ TILs showed significantly better overall survival (*p* = 0.028), in multivariate analysis, independently of tumour stage and histological subtype. There are no studies on ITAC to compare our results with; however, in other sinonasal tumour types such as SNSCC and olfactory neuroblastoma (ONB), CD8^+^ TILs have also been found to correlate to better survival [18,19]. The same has been reported in head and neck cancer [26,27], lung adenocarcinoma [28,29] and most other studied cancers. This confirms the protective, cytotoxic anti-tumour function of CD8^+^ TILs. 

Our evaluation of TMIT classification showed that type I and IV cases (CD8^high^ and eitherPD-L1^+^ or PD-L1^-^) were exclusively papillary and colonic and had much better overall survival. This indicates that the more important component of TMIT is an elevated presence of CD8^+^ TILs, while tumoural PD-L1 expression does not have additional prognostic value. Nevertheless, among the CD8^low^ cases, those expressing PD-L1 showed a tendency toward a worse survival (Figure 3). This confirms previous studies on PD-L1 in sinonasal tumours of different histotypes [17,18]. Also for lung adenocarcinomas, a tumour type similar to ITAC, most studies report a worse survival in PD-L1-positive cases [29,30], which may be explained by PD-L1 immunosuppressive effect, which prevents cytolysis by activated CD8^+^ TILs.

We observed PD-L1- and CD68-positive macrophages in the stromal compartment in 17% (23/133) of cases, which significantly co-occurred with CD8^+^ TILs. Infiltration of macrophages in solid tumours has been associated with poor prognosis, promoting angiogenesis and invasion, and suppressing antitumour immunity [31]. These anti-inflammatory and pro-tumoural properties are mostly ascribed to M2 (CD68^+^ and CD163^+^/CD204+) macrophages, whereas M1 macrophages (CD68^+^ and HLA-DR^+^) would be pro-inflammatory [32,33]. However, this distinction is not generally accepted, and both M1 and M2 macrophage infiltrations have also been associated with favourable clinical outcome, perhaps through the recruitment of CD8^+^ TILs [34,35,36]. In our study, the association of CD68-positive macrophages with CD8^+^ TILs and a prolonged overall survival appears to support an antitumour role for both types of infiltrating lymphocytes.

Immune checkpoint inhibitors are an emerging and hopeful therapeutic option for a variety of tumours, and several clinical trials are showing good efficacy and tolerance to PD-1 checkpoint inhibitors in locally advanced HNSCC [14,15,16]. However, clinical responses are seen in only 13–22% of patients, underlining the need for predictive biomarkers. The presence of tumour-activated CD8^+^ TILs is an essential requirement for the treatment with immune checkpoint inhibitors, while PD-L1 expression has been shown to be associated with improved outcomes upon immunotherapy [24]. These two factors are united in the TMIT classification, where type I (CD8^high^/PD-L1+) and, to a lesser extent, type IV (CD8^high^/PD-L1-) tumours are predicted to present the best response [22,23,24]. Our analysis revealed 8/133 (6%) of ITAC to be TMIT I, and 1.5% TMIT IV and indicated that ITAC is a lowly immunogenic cancer, with solid and mucinous subtypes even less immunogenic. This contrasts with known strong immunogenic tumours such as skin melanoma, renal cell and bladder cancer or head and neck and lung SCC, where TMIT I characterizes 40–50% of cases [21]. On the other hand, ITAC is comparable to adenocarcinomas of other organs such as lung, colon and stomach, with reports of 12–14%, 19% and 23% TMIT I, respectively [21,28,37,38].

A limitation of using TMIT as a predictor of response to therapy is the lack of consensus on the scoring and cut-off values of CD8^+^ TILs and PD-L1. Indeed, the proportions of cases among the TMIT types vary considerably between studies on the same types of cancer. In addition, the exact localization of TILs, either inside the tumour or in the stroma, can be important. Moreover, the studied series of tumours may be heterogeneous, for example, squamous cell carcinoma and adenocarcinoma subtypes in non-small cell lung cancer. Similarly, ITAC includes different histological subtypes, and we found significant differences among them. Among the mucinous tumours, none were TMIT I or IV, and PD-L1-positive macrophages were virtually absent. This suggests that this subtype is even less immunogenic than papillary and colonic ITAC. Another possible limitation of our study is the large period of time over which the samples were collected. To address this, we compared cases treated before and after the year 2003, the point in time when endoscopic surgery was introduced in our hospital, and we found the same distribution of CD8^+^ TILs and TMIT types and the same association with overall survival. Apart from CD8^+^ TILs, the tumour microenvironment includes many more types of immune cells (myeloid-derived suppressor cells, regulatory T cells, dendritic cells, macrophages, natural killer cells, B cells) that may also be related to prognosis or play a role in the response to immune therapy [32]. However, from all studies, the strongest relation to clinical outcome and therapeutic response are found for CD8^+^ TILs [23,39]. Finally, aside from CD8^+^ TILs, PD-L1 expression and TMIT, there are other biomarkers that may predict the efficacy of anti-PD1 immunotherapy, such as tumour mutational burden, lactate dehydrogenase concentration in serum and pro-inflammatory interferon gene signature [24].

## 5. Conclusions

The main aim of this study was to evaluate possible predictors of response to immunotherapy. Our results demonstrate that intratumoural CD8^+^ TILs are present in up to 65% of ITAC. Moreover, a high number of CD8^+^ TILs and TMIT types I and IV were associated with longer overall survival. TMIT classification did not have additional prognostic value over CD8+ TILs alone. The modest percentage of TMIT type I CD8^high^/PD-L1^pos^ cases indicate that ITAC is a lowly immunogenic tumour type. Nevertheless, a subgroup of patients may benefit from anti-PD-1 agents already approved in HNSCC.

## Figures and Tables

**Table 1:** Clinical characteristics according to CD8^+^ tumour-infiltrating lymphocytes (TILs) and tumour microenvironment immune type (TMIT) type.

Clinical Features	All	CD8+ TI Ls				TMIT Type				
		0%	1–10%	>10%	Significance	I	II	III	IV	Significance
All	133	47	76	10		8	96	27	2	
Gender					0.467					0.854
Female	2 (2)	0 (0)	2 (3)	0 (0)		0 (0)	2 (2)	0 (0)	0 (0)	
Male	131 (98)	47 (100)	74 (97)	10 (100)		8 (100)	94 (98)	27 (100)	2 (100)	
Disease stage					0.696 a					0.776 a
I	30 (22)	12 (25)	18 (23)	0 (0)		0 (0)	22 (23)	8 (30)	0 (0)	
II	17 (13)	5 (11)	8 (11)	4 (40)		4 (50)	13 (14)	0 (0)	0 (0)	
III	45 (34)	16 (34)	25 (33)	4 (40)		2 (25)	31 (32)	10 (37)	2 (100)	
IV-a	16 (12)	8 (17)	6 (8)	2 (20)		2 (25)	10 (10)	4 (15)	0 (0)	
IV-b	25 (19)	6 (13)	19 (25)	0 (0)		0 (0)	20 (21)	5 (18)	0 (0)	
Histological type					0.007 b					0.188 b
Papillary	13 (10)	3 (6)	9 (12)	1 (10)		1 (13)	8 (8)	4 (15)	0 (0)	
Colonic	80 (60)	23 (49)	48 (63)	9 (90)		7 (87)	56 (59)	15 (55)	2 (100)	
Solid	10 (7)	3 (6)	7 (9)	0 (0)		0 (0)	6 (6)	4 (15)	0 (0)	
Mucinous	30 (23)	18 (39)	12 (16)	0 (0)		0 (0)	26 (27)	4 (15)	0 (0)	
Recurrence					0.165					0.079
No	70 (53)	20 (43)	43 (57)	7 (70)		6 (75)	44 (46)	19 (70)	1 (50)	
Yes	63 (47)	27 (57)	33 (43)	3 (30)		2 (25)	52 (54)	8 (30)	1 (50)	
Metastasis					0.249					0.389
No	120 (90)	40 (85)	70 (92)	10 (100)		8 (100)	84 (88)	26 (96)	2 (100)	
Yes	13 (10)	7 (15)	6 (8)	0 (0)		0 (0)	12 (12)	1 (4)	0 (0)	
Patient status					0.021 c					0.054 c
Alive	60 (45)	20 (43)	32 (42)	8 (80)		6 (75)	42 (44)	10 (37)	2 (100)	
Died of disease	53 (40)	19 (40)	34 (45)	0 (0)		0 (0)	42 (44)	11 (41)	0 (0)	
Died other causes	20 (15)	8 (17)	10 (13)	2 (20)		2 (25)	12 (12)	6 (22)	0 (0)	

a: Chi^2^ analysis comparing disease stages I, II, III with IVa, IVb; b: Chi^2^ analysis comparing papillary-colonic with solid-mucinous intestinal-type adenocarcinoma (ITAC); c: Chi^2^ analysis comparing patients alive with patients who died of the disease.

**Table 2:** Multivariate Cox regression survival analysis of tumour stage, histological type and CD8^+^ TILs.

	Univariate		Multivariate	
	Log Rank	Significance	HR (95% CI)	Significance
Tumour stage (stages I–III versus IVa, IVb)	50.498	*p* = 0.000	5.15 (3.06–8.70)	*p* = 0.000
Histological type (papillary-colonic versus solid-mucinous)	15.085	*p* = 0.002	1.21 (0.73–2.03)	*p* = 0.461
CD8^+^ TILs (negative/low versus high)	4.829	*p* = 0.028	0.16 (0.04–0.67)	*p* = 0.012

HR: Hazard ratio; CI: Confidence interval.

**Table 3:** Correlations between CD8^+^ TILs and PD-L1-expressing tumour cells and macrophages.

		PD-L1 Expression Tumour Cells			PD-L1 Expression Macrophages		
		Negative	Positive	Significance	Negative	Positive	Significance
CD8^+^ TILs	0%	41	6		45	2	
	1–10%	55	21	*p* = 0.000	60	16	*p* = 0.001
	>10%	2	8		5	5	

## Supplementary Materials

The following are available online at https://www.mdpi.com/2076-393X/8/2/202/s1, Supplementary Table S1. Clinical characteristics according to PD-L1^+^ macrophages.
