# Supplementary material for: CD8+ Tumour-Infiltrating Lymphocytes and Tumour Microenvironment Immune Types as Biomarkers for Immunotherapy in Sinonasal Intestinal-Type Adenocarcinoma
Source: Vaccines (Basel). 2020 Apr 28;8(2):202. doi: 10.3390/vaccines8020202 (PMC7349388; doi:10.3390/vaccines8020202)

**Supplementary Material**

**Supplementary Table 1.** Clinical characteristics according to PD-L1^+^ macrophages and CMTM6 expression.

|  |  | **PD-L1^+^ Macrophages** | | | **CMTM6 expression** | | | |
| --- | --- | --- | --- | --- | --- | --- | --- | --- |
|  |  |  |  |  |  |  |  |  |
|  | all | no | yes | significance | negative | low | high | significance |
|  | 133 | 110 | 23 |  | 72 | 38 | 23 |  |
| Gender |  |  |  | 0.317 |  |  |  | 0.423 |
| Female | 2 (2) | 1 (1) | 1 (4) |  | 2 (3) | 0 (0) | 0 (0) |  |
| Male | 131 (98) | 109 (99) | 22 (96) |  | 70 (97) | 38 (100) | 23 (100) |  |
| Disease stage |  |  |  | 0.804^a^ |  |  |  | 0.149^a^ |
| I | 30 (22) | 23 (21) | 7 (31) |  | 13 (18) | 11 (29) | 6 (26) |  |
| II | 17 (13) | 13 (12) | 4 (17) |  | 8 (11) | 6 (16) | 3 (13) |  |
| III | 45 (34) | 39 (35) | 6 (26) |  | 24 (33) | 11 (29) | 10 (43) |  |
| IV-a | 16 (12) | 15 (14) | 1 (4) |  | 9 (13) | 5 (13) | 2 (9) |  |
| IV-b | 25 (19) | 20 (18) | 5 (22) |  | 18 (25) | 5 (13) | 2 (9) |  |
| Histological type |  |  |  | 0.211^b^ |  |  |  | 0.331^b^ |
| Papillary | 13 (10) | 11 (10) | 2 (9) |  | 6 (8) | 5 (13) | 2 (9) |  |
| Colonic | 80 (60) | 63 (57) | 17 (74) |  | 41 (57) | 25 (66) | 14 (61) |  |
| Solid | 10 (7) | 7 (6) | 3 (13) |  | 6 (8) | 2 (5) | 2 (9) |  |
| Mucinous | 30 (23) | 29 (27) | 1 (4) |  | 19 (27) | 6 (16) | 5 (21) |  |
| Recurrence |  |  |  | 0.819 |  |  |  | 0.003 |
| No | 70 (53) | 57 (52) | 13 (56) |  | 30 (42) | 21 (55) | 19 (83) |  |
| Yes | 63 (47) | 53 (48) | 10 (44) |  | 42 (58) | 17 (45) | 4 (17) |  |
| Metastasis |  |  |  | 0.698 |  |  |  | 0.210 |
| No | 120 (90) | 100 (91) | 20 (87) |  | 63 (87) | 34 (89) | 23 (100) |  |
| Yes | 13 (10) | 10 (9) | 3 (13) |  | 9 (13) | 4 (11) | 0 (0) |  |
| Patient status |  |  |  | 0.623^c^ |  |  |  | 0.134^c^ |
| Alive | 60 (45) | 48 (44) | 12 (52) |  | 28 (39) | 19 (50) | 13 (56) |  |
| Died of disease | 53 (40) | 45 (41) | 8 (35) |  | 33 (46) | 15 (39) | 5 (22) |  |
| Died other causes | 20 (15) | 17 (15) | 3 (13) |  | 11 (15) | 4 (11) | 5 (22) |  |

**Supplementary Figure 1.** Immunohistochemical staining of CMTM6 showing absence (A), low (B) and high (C) expression.


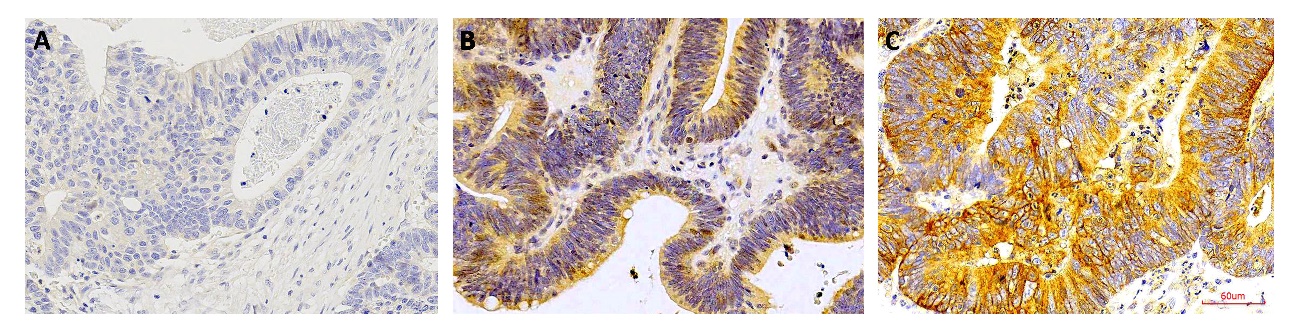


**Supplementary Figure 2.** Wildtype sequence of part of exon 6 of the PD-L1 gene including the DTNSK motif.


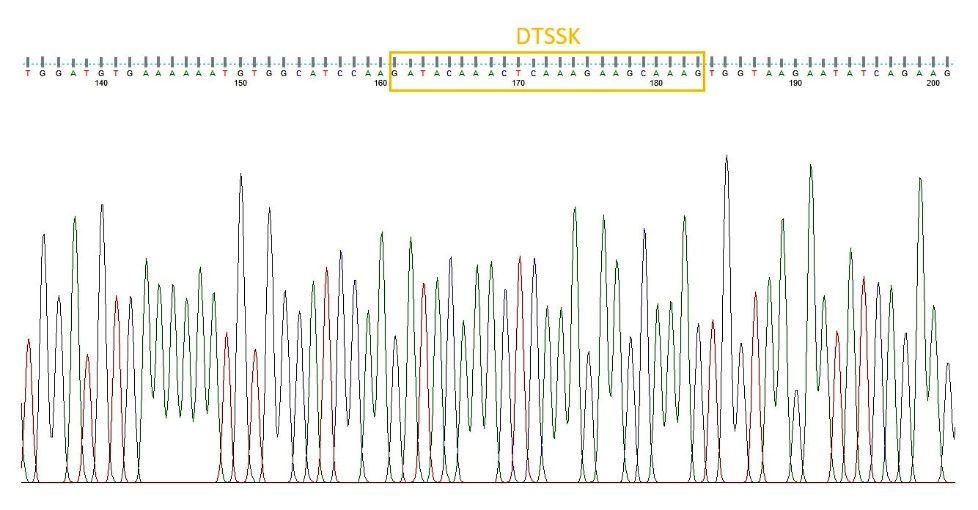

Supplement: Supplementary file 1 [file vaccines-08-00202-s001.zip › vaccines-781264-supplementary.docx]
